# The Effect of Aging on Retinal Function and Retinal Ganglion Cell Morphology Following Intraocular Pressure Elevation

**DOI:** 10.3389/fnagi.2022.859265

**Published:** 2022-05-12

**Authors:** Pei Ying Lee, Da Zhao, Vickie H. Y. Wong, Vicki Chrysostomou, Jonathan G. Crowston, Bang V. Bui

**Affiliations:** ^1^Department of Optometry and Vision Sciences, University of Melbourne, Parkville, VIC, Australia; ^2^Singapore Eye Research Institute, Singapore, Singapore; ^3^Duke-NUS Medical School, Singapore, Singapore

**Keywords:** aging, intraocular pressure, retinal ganglion cells, recovery, electroretinogram, morphology

## Abstract

Aging and elevated intraocular pressure (IOP) are two major risk factors for glaucomatous optic neuropathy; a condition characterized by the selective, progressive injury, and subsequent loss of retinal ganglion cells (RGCs). We examined how age modified the capacity for RGCs to functionally recover following a reproducible IOP elevation (50 mmHg for 30 min). We found that RGC functional recovery (measured using electroretinography) was complete by 7 days in 3-month-old mice but was delayed in 12-month-old mice until 14 days. At the 7-day recovery endpoint when RGC function had recovered in young but not older eyes, we examined RGC structural responses to IOP-related stress by analyzing RGC dendritic morphology. ON-RGC cell volume was attenuated following IOP elevation in both young and older mice. We also found that following IOP elevation OFF-RGC dendritic morphology became less complex per cell volume in young mice, an effect that was not observed in older eyes. Our data suggest that adaptations in OFF-RGCs in young eyes were associated with better functional recovery 7 days after IOP elevation. Loss of RGC cellular adaptations may account for delayed functional recovery in older eyes.

## Graphical Abstract

Retinal ganglion cell (RGC) morphological changes 7 days after intraocular pressure (IOP) elevation to 50 mmHg for 30 min.

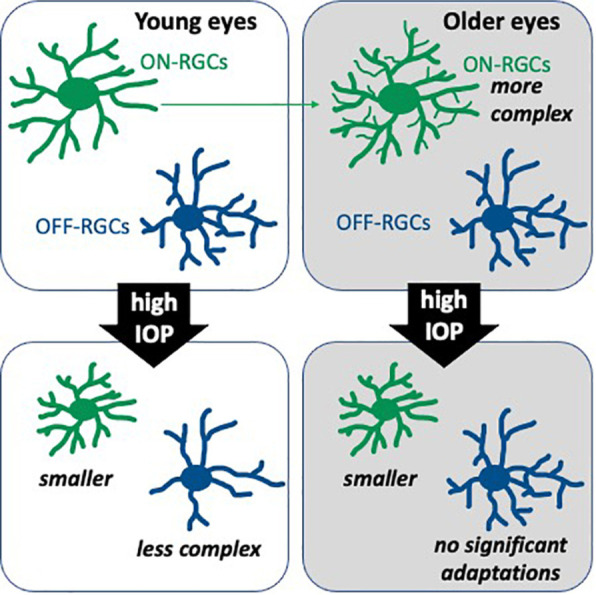

## Introduction

Retinal ganglion cells (RGCs) are retinal neurons that convey visual information to the brain *via* long axons (Sanes and Masland, 2015). RGC dendrites receive synaptic input from neighboring bipolar cells, which receive information from photoreceptors, and amacrine cells (Agostinone and Di Polo, 2015; Sanes and Masland, 2015). Progressive loss of RGCs is a well-characterized feature of glaucomatous optic neuropathy, with aging and elevated intraocular pressure (IOP) documented as the two major risk factors (Quigley, 2011; Weinreb et al., 2014; Jonas et al., 2017). Accumulating clinical and experimental studies have shown that the functional loss may at least in the short-term be reversible as IOP lowering and nicotinamide have been shown to improve inner retinal function (Fry et al., 2018; Zhao et al., 2019; Hui et al., 2020; De Moraes et al., 2021). There appears to be a period of RGC adaptation and dysfunction that precedes irreversible cell apoptosis (Calkins, 2012, 2021). Whilst there is ample evidence that RGCs show functional and structural adaptations to IOP-related stress, our understanding of how aging impacts this functional adaptive capacity is limited (Di Pierdomenico et al., 2021).

Lim et al. (2014) showed that after an hour of stepwise IOP elevation from 10 to 100 mmHg (3-min duration for each 5-mmHg step), retinal function measured using light-adapted electroretinogram (ERG) recovered to baseline level within 2 h in 3-month-old rats, whereas function was still attenuated in 14-month-old rats after 2 h. Seven days after a controlled, sub-ischemic IOP elevation (50 mmHg for 30 min), RGC function assessed using scotopic full-field ERG recovered in 3-month-old, but not 12- and 18-month-old C57BL/6 mice (Kong et al., 2012; Chrysostomou and Crowston, 2013). Older mice (12-month-old) did not show full functional recovery until 28 days after (Crowston et al., 2017). These findings point to a failure of adaptive mechanisms in aging, which may increase the risk of irreversible injury from repeated IOP challenge in glaucoma.

Previous studies suggest that dendritic remodeling is one of the earliest structural changes that occurs in RGCs in response to stress, well before cell death (Della Santina and Ou, 2016). There is also growing evidence for RGC type-specific vulnerability to injury, including NMDA excitotoxicity, optic nerve crush and IOP elevation (Della Santina and Ou, 2017; Christensen et al., 2019; Wang et al., 2019; Yang et al., 2020; Zhang et al., 2022). Current literature has identified and validated at least 30 types of RGCs (Sanes and Masland, 2015; Baden et al., 2016; Bae et al., 2018). Of those, studies investigating RGC changes in response to IOP elevation have mostly examined ON or OFF RGCs, which correspond to their receptive field preferences for increments or decrements to light, respectively, (Kuffler, 1953; Della Santina et al., 2013; El-Danaf and Huberman, 2015; Ou et al., 2016; Risner et al., 2018). The dendrites of ON RGCs synapse with ON bipolar cells in the inner region of the inner plexiform layer, whereas those of OFF RGCs synapse with OFF bipolar cells in the distal region of the inner plexiform layer (Wang et al., 2019). Some studies have reported that in response to chronic IOP elevation, dendritic fields of ON-RGCs in young mice become smaller and less complex (Feng et al., 2013, 2016; Bhandari et al., 2019). However, other studies suggest that OFF-RGCs are more vulnerable, showing reduced dendritic complexity (Della Santina et al., 2013; El-Danaf and Huberman, 2015; Ou et al., 2016). These studies employed more severe and/or more chronic periods of IOP elevation, which could lead to RGC death, making the earliest adaptations more difficult to study. Moreover, modeling injury in young eye has left a gap in our understanding of how aging might impact RGC functional and morphological adaptations to stress.

Thy1-YFP transgenic mice have been used in glaucoma studies as they express fluorescent neurons under the control of Thy1 promoter, a glycoprotein found on the surface of neurons including RGCs (Barnstable and Drager, 1984; Feng et al., 2000). In particular, Thy1-YFPh mice have as few as 0.2%, up to 10%, of total RGCs fluoresce (Feng et al., 2000; Oglesby et al., 2012; Iaboni et al., 2020). This spareness, often with little or no overlap between fluorescent RGCs, allows the full extent of RGC dendritic fields to be quantified in response to elevated IOP in young and older eyes (Coombs et al., 2006; Li et al., 2011; Della Santina et al., 2013; Feng et al., 2013; Iaboni et al., 2020).

The current study systematically examined the time course of *in vivo* functional recovery from a well-controlled sub-ischemic IOP elevation (50 mmHg for 30 min) in both 3- and 12-month-old C57BL/6 and Thy1-YFPh mice. We tested the hypothesis that in response to IOP elevation, older eyes showed slower functional recovery and ON- and OFF-RGCs adapted differently with age.

## Materials and Methods

### Animals

All experimental procedures conducted adhered to the *Australian code for the care and use of animals for scientific purposes* set out by National Health and Medical Research Council and the Association for Research in Vision and Ophthalmology’s Statement for the Use of Animals in Ophthalmic and Vision Research. Ethics approval was obtained from The Florey Institute of Neuroscience and Mental Health Animal Ethics Committee (17-028-UM).

Male and female C57BL/6JArc mice (Animal Resource Centre, Canning Vale, WA, Australia) and B6.Cg-Tg(Thy1-YFP)HJrs/J mice (Thy1-YFPh for short), aged 3–4 and 12–14 months were used. The Thy1-YFPh colony was established with breeder pairs (The Jackson Laboratory, Bar Harbor, ME, United States) and C57BL/6J mice, and was bred at The Florey Institute for Neuroscience and Mental Health (Parkville, VIC, Australia). Genotyping was done using tail samples through Transnetyx (Cordova, TN, United States).

All mice were housed with unrestricted access to standard lab chow (Barastoc mouse pellet, Ridley Corporation, Melbourne, VIC, Australia) and water at the University of Melbourne (Kenneth Myer Building, Parkville, VIC, Australia). The animal facility was well ventilated and maintained at 21^°^C. To maintain diurnal regularity of IOP and to reduce light-induced retinal injury, room lighting at the facility was kept on a 12-h light/dark cycle (lights on 7 am) and below 50 lux (Penn et al., 1987; Organisciak et al., 1998, 2000; Aihara et al., 2003).

Prior to procedures, animals were anesthetized with ketamine:xylazine (80:10 mg/kg, Troy Laboratories, Glendenning, NSW, Australia) delivered intraperitoneally. Topical proxymetacaine (0.5% Alcaine, Alcon Laboratories, French Forests, NSW, Australia) and tropicamide (1% Mydriacyl, Alcon Laboratories) were instilled to induce local anesthesia and pupil dilation, respectively. Animal core body temperature was maintained at 37.5 ± 0.5^°^C on a heated platform throughout experiments.

### Intraocular Pressure Elevation

We have developed a highly reproducible, minimally invasive RGC stress model that leads to temporary loss of inner retinal function and only low levels of RGC loss (<5 to 10%; Crowston et al., 2015). A glass needle (inner diameter of tip ∼50 μm, created from borosilicate glass capillaries 1B100-6, World Precision Instruments, Sarasota, FL, United States) was inserted into the anterior chamber. The needle was connected through a pressure transducer (Transpac IV; Abbott Critical Care Systems, Sligo, Ireland) to a syringe on a motorized syringe pump (Pump 11 Plus or Standard Infuse/Withdraw Pump 11 Elite Programable Syringe Pump, Harvard Apparatus, Holliston, MA, United States), which was used to infuse Hanks’ Balanced Salt solution (H6648, Sigma-Aldrich, North Ryde, NSW, Australia) into the anterior chamber. IOP was elevated to 50 mmHg for 30 min (Kong et al., 2009; Crowston et al., 2015) and was monitored real time through the transducer connected to a PowerLab data acquisition system (PowerLab/8SP, ADInstruments, Bella Vista, NSW, Australia). Supplementary Figure 1 shows an example of the IOP tracked on the LabChart software (ADInstruments). The contralateral eye served as an untreated control. Recovery of retinal function from IOP elevation was examined at different experimental endpoints (each a separate group of animals at 3, 7, 14, or 28 days) for each age group in both strains.

### Electroretinography

Retinal function was assessed using dark-adapted full-field ERG as described previously (Zhao et al., 2017). Mice were dark adapted overnight, and all animal preparation was conducted under dim red light in a darkened lightproof room to maximize retinal sensitivity and optimize RGC-mediated scotopic threshold response measurement (Saszik et al., 2002; Bui and Fortune, 2004). The active and reference electrodes, made of silver wire (99.9% pure, A&E Metal Merchants, Sydney, NSW, Australia) connected to platinum leads (F-E2, Grass Technologies, West Warwick, RI, United States), were placed on the central cornea and sclera (around the equator), respectively. A stainless-steel needle (F-E2, Grass Technologies), inserted subcutaneously into the tail, served as the ground electrode to reduce noise. Responses from both eyes were measured simultaneously. Animals were kept warm using a water-heated platform during ERG recording.

Electroretinography responses were elicited using a series of progressively increasing luminous energies (–6.35 to 2.07 log cd.s.m^–2^) delivered by a pre-calibrated (IL1700, International Light Technologies, Peabody, MA, United States) LED light source embedded into a Ganzfeld sphere (Photometric Solutions International, Huntingdale, VIC, Australia). This range of stimulus energies elicits signals for the key neuronal classes in the retina. The first electronegative component of the ERG waveform (the a-wave) elicited by the brightest stimuli was modeled using a delayed-Gaussian function (P3 model) to expose the maximal photoreceptor response (RmP3 amplitude; Lamb and Pugh, 1992). The large positive deflection of the raw ERG waveform (the *b*-wave) is a composite of the corneal negative photoreceptor response (P3) and the positive bipolar cell response (P2). The modeled P3 was subtracted from the raw ERG waveform to reveal the maximum amplitude of the positive component (*V*_*max*_), which is a measure of bipolar cell integrity (Naka and Rushton, 1966; Fulton and Hansen, 1988). The RGC response was elicited at very dim luminous energies near the absolute visual threshold, measured as the positive scotopic threshold response (pSTR; Saszik et al., 2002; Bui and Fortune, 2004).

### Optical Coherence Tomography

Following ERG recording, mice were placed on a heated platform and a thick lubricating gel (GenTeal Gel, hypromellose 0.3%/carbomer 980 0.22%, Alcon Laboratories) was applied onto the cornea, with a glass coverslip placed on top, to help clear the reversible cataracts induced by general anesthesia. Once the cataract was clear, mice were positioned on a 3-dimensional adjustable platform where the corneal apex was aligned to the objective lens of the Spectralis imaging system (Heidelberg Engineering GmbH, Heidelberg, Germany). Retinal optical coherence tomography (OCT) was taken for each eye, centered at the optic disk (volume scan of 30^°^ × 25^°^, 8.0 mm × 6.7 mm, 768 A/B-scan, 121B-scans). The OCT images were processed within the Spectralis system to segment each retinal layer automatically using the in-built segmentation algorithm (HEYEX v6.16.2, Heidelberg Engineering). The thicknesses of the 4 regions (superior, inferior, nasal, and temporal) in the outer ring (3–6 mm) of an Early Treatment Diabetic Retinopathy Study (ETDRS) grid centered at the optic disk were averaged. As the boundary between the ganglion cell layer and the inner plexiform layer of the mouse retina can be difficult to distinguish, these two layers were combined and presented as the ganglion cell – inner plexiform layers.

### Immunohistochemistry

Following *in vivo* assessment, eyes were collected and fixed in 4% paraformaldehyde in 0.01M phosphate buffered saline (PBS) for an hour at room temperature, and then stored in PBS at 4^°^C. In this study, 16 eyes from the 7-day recovery endpoint group (*n* = 4 Thy1-YFPh mice for each age group) were dissected. This time point was chosen as a clear difference in functional recovery between young and older mice was observed.

Retinae were isolated and then rinsed 3 times (10 min each) with PBS, followed by incubation with goat anti-green fluorescent protein antibody (1:400, Rockland Immunochemicals 600-141-215, Limerick, PA, United States) overnight at room temperature to enhance the intrinsic fluorescent RGCs. Following this, retinae were rinsed 3 times with PBS and left in clean PBS for an hour at room temperature. They were then counterstained with a nuclear stain (1:1000, Hoechst, Thermo Fisher Scientific, Scoresby, VIC, Australia) for 10 min at room temperature. Following 3 rinses with PBS, retinae were flat mounted with RGC side up, using Dako fluorescence mounting medium (Agilent Technologies, Santa Clara, CA, United States).

Retinal samples were imaged with the LSM 880 confocal microscope (Carl Zeiss, North Ryde, NSW, Australia) using a 20× objective. *Z*-stack images for RGC morphology were taken with Airyscan Fast super-resolution mode at 1.7× zoom (voxel size: 0.0744 × 0.0744 × 0.397 μm). Fluorescent RGCs with a clear axon, showing little to no overlapping dendrites with neighboring RGCs, were randomly chosen from various retinal quadrants for imaging (4–7 cells per retina). Depending on the size of the cell, a tile scan (up to 2 × 3 or 3 × 2) was required to image the full dendritic extent of the RGC. Due to the large file sizes and length of imaging time for RGC morphology, a combined RGC morphology and Hoechst (used as retinal depth reference) channels were imaged separately in the same session using the fast scan mode (voxel size: 0.247 × 0.247 × 0.342 μm).

Images were analyzed using the Imaris software (Bitplane AG, Oxford Instruments, Zurich, Switzerland). Following automatic tracing using the in-built Filament tool, manual tracing was completed for all RGCs (24 cells per treatment per age group) using the Edit and AutoPath tracing tools to outline the full morphology of the cell. Four parameters describing cell morphology were extracted from Imaris: (1) Sholl intersections – the number of dendritic intersections at each 5 μm distance from the soma, (2) total dendritic length – the total length of all dendrites combined (μm), (3) number of dendritic branches – the total number of dendrite segments, and (4) convex hull volume – the volume enclosing the dendritic arbor bounded by joining the ends of each dendritic terminal (μm^3^). Further information extracted from the Sholl intersections output included: (1) area under the curve (AUC) – area under the Sholl profile, (2) peak number of intersections – the highest number of intersections within the Sholl profile, and (3) peak position – the distance from the cell soma to where the peak number of intersections was located (μm). RGCs were also further classified as ON or OFF cells if they stratified within 0–40% and 60–100% of the inner plexiform layer depth from the ganglion cell layer, respectively, (Coombs et al., 2007; Wang et al., 2021). The extent of the inner plexiform layer was defined using the Hoechst channel to demarcate the inner nuclear layer.

### Statistical Analysis

Data were collected and analyzed with experimenters masked to IOP treatment and age. Group data are presented as group mean ± standard error of the mean (SEM). All ERG data normalized to contralateral control eyes were analyzed using a two-way ANOVA to compare recovery endpoint and age (Prism 8, GraphPad Software, San Diego, CA, United States). For RGC morphology analysis, the comparison of 3- and 12-month-old control eyes was conducted using unpaired two-tailed *t*-tests. Analyses involving IOP treatment with regards to cell type or age were done using mixed effect models in Minitab 19 (Minitab, LLC, State College, PA, United States). Area under the curve (an index of cell complexity) and convex hull volume (an index of cell size) were combined in a linear regression to compare slope differences between age groups or treatment (Prism 8). Linear regressions were plotted with their 95% confidence interval.

## Results

### Functional Recovery in C57BL/6 Mice

**FIGURE 1:**
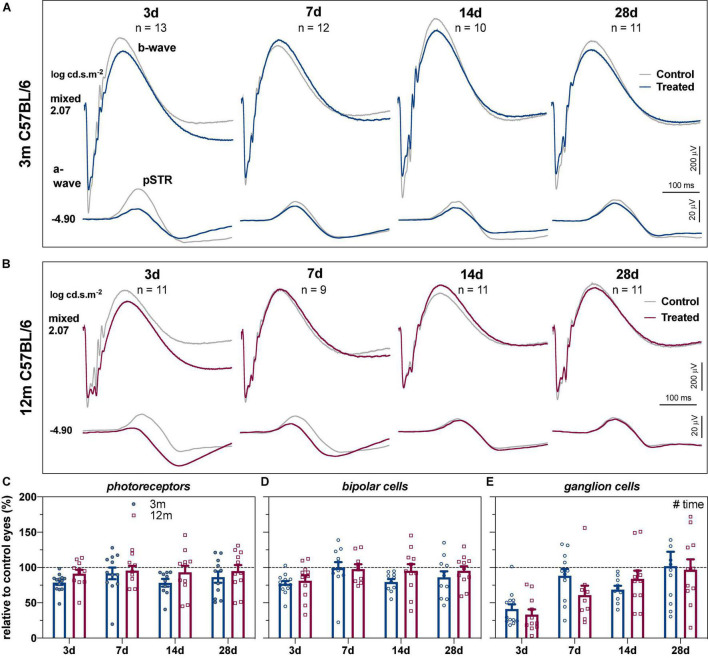
Recovery of retinal function in 3- and 12-month-old C57BL/6 mice. **(A)** Group averaged ERG waveforms in 3-month-old mice (blue traces, treated eyes; gray traces, control eyes) at luminous energies representing predominantly ganglion cell responses (–4.90 log cd.s.m^–2^) and outer retinal function (2.07 log cd.s.m^–2^) at 3 (*n* = 13), 7 (*n* = 12), 14 (*n* = 10), and 28 days (*n* = 11). **(B)** Group averaged ERG waveforms in 12-month-old mice (red traces, treated eyes; gray traces, control eyes) at 3 (*n* = 11), 7 (*n* = 9), 14 (*n* = 11), and 28 days (*n* = 11). **(C–E)** ERG responses from IOP-treated eyes expressed relative to their contralateral control eyes for **(C)** photoreceptor amplitude, **(D)** bipolar cell amplitude, and **(E)** ganglion cell amplitude (pSTR) in 3-month-old (blue bars) and 12-month-old (red bars) mice. Error bars, SEM; #, two-way ANOVA *p* < 0.05.

The group averaged ERG waveforms in response to 2 selected stimulus energies representing predominantly RGC (–4.90 log cd.s.m^–2^) and outer retinal responses (2.07 log cd.s.m^–2^) at the 4 recovery endpoints for 3- and 12-month-old C57BL/6 mice are summarized in Figure 1. In 3-month-old C57BL/6 mice (Figure 1A), 3 days after acute IOP elevation there was a reduction in the pSTR (RGC mediated responses), with less dysfunction in the outer retinal b-wave and a-wave (bipolar cell and photoreceptor mediated responses, respectively). At later recovery endpoints, differences between control and IOP-treated eyes were more subtle in young eyes. In 12-month-old mice, the pSTR was reduced in IOP-treated eyes compared to control eyes at 3 and 7 days after IOP elevation. Like young eyes, there was little dysfunction in outer retinal function compared with the pSTR (Figure 1B).

To address the variability between groups at the different recovery endpoints and to facilitate comparison between age groups, retinal responses were expressed relative to their contralateral control eyes. Two-way ANOVA confirmed that there was no difference in recovery endpoints (time) between the two ages for photoreceptor (Figure 1C, age, *F*_1_,_80_ = 3.97, *p* = 0.050; time, *F*_3_,_80_ = 0.653, *p* = 0.584) or bipolar cell amplitudes (Figure 1D, age, *F*_1_,_80_ = 1.87, *p* = 0.175; time, *F*_3_,_80_ = 2.51, *p* = 0.065). In contrast, there was a significant recovery endpoints effect for RGC amplitude with gradual recovery of RGC function occurring over time (Figure 1E, *F*_3_,_80_ = 9.74, *p* < 0.001).

**FIGURE 2:**
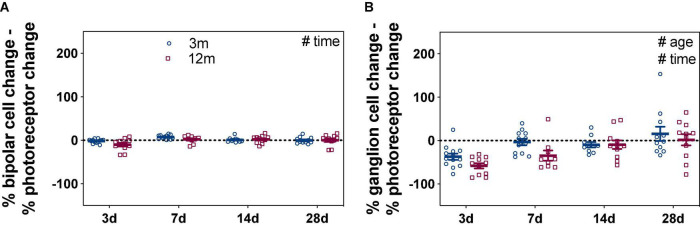
Bipolar cell and ganglion cell responses expressed relative to their own photoreceptor input. Responses from IOP-treated eyes were expressed relative to their contralateral control eyes (%). **(A)** For each eye, the % photoreceptor response was subtracted from % bipolar cell response. This normalizes the bipolar cell response to their photoreceptor input. **(B)** For each eye, the % photoreceptor response was subtracted from % ganglion cell response. This normalizes the ganglion cell response to their photoreceptor input. A resultant negative % difference indicates that ganglion cell function was more affected than expected from photoreceptor input. Error bars, SEM; #, two-way ANOVA *p* < 0.05.

As ERG components are generated in a series, deficits in photoreceptor function could lead to reduced input into bipolar cells and RGCs (Nguyen et al., 2013). One way to account for this is to express inner retinal ERG components relative to the photoreceptor response (Figure 2); specifically, the percentage differences between photoreceptor and bipolar cell amplitudes (% change), as well as between photoreceptor and RGC amplitudes (% change), were quantified. Figure 2A shows any difference in bipolar cell could be accounted for by the expected photoreceptor input. Two-way ANOVA revealed a significant time effect (*F*_3_,_80_ = 5.93, *p* = 0.001) with no difference between ages (*F*_1_,_80_ = 2.69, *p* = 0.105).

Comparison between the percentage loss of photoreceptor and RGC responses was more telling, resulting in a significant age (Figure 2B, *F*_1_,_80_ = 5.11, *p* = 0.027) and time effects (*F*_3_,_80_ = 11.17, *p* < 0.001). At 3 days after acute IOP elevation, 3-month-old mice had RGC responses that were –37.3 ± 7.0% worse than expected from their photoreceptor input. By 7 days, RGC responses were close to that expected from photoreceptor input (–3.4 ± 7.4%) and remained so after 14 (–9.7 ± 6.0%) and 28 (15.6 ± 16.4%) days of recovery from IOP elevation. In contrast to young eyes, there was slower inner retinal recovery in 12-month-old mice. RGC responses were more affected than expected from photoreceptor responses at 3 (–58.1 ± 6.1%) and 7 (–34.7 ± 11.7%) days. Only after 14 (–9.4 ± 10.0%) and 28 (1.9 ± 13.1%) days of recovery from acute IOP elevation were RGC responses similar to that expected from their photoreceptor input in older eyes. These results show that acute IOP elevation affected RGCs more, with older eyes taking a week longer to fully recover.

Comparison of IOP-treated eyes to their own baseline ERG measurements showed similar findings (Supplementary Figure 2).

### Functional Recovery in Thy1-YFPh Mice

**FIGURE 3:**
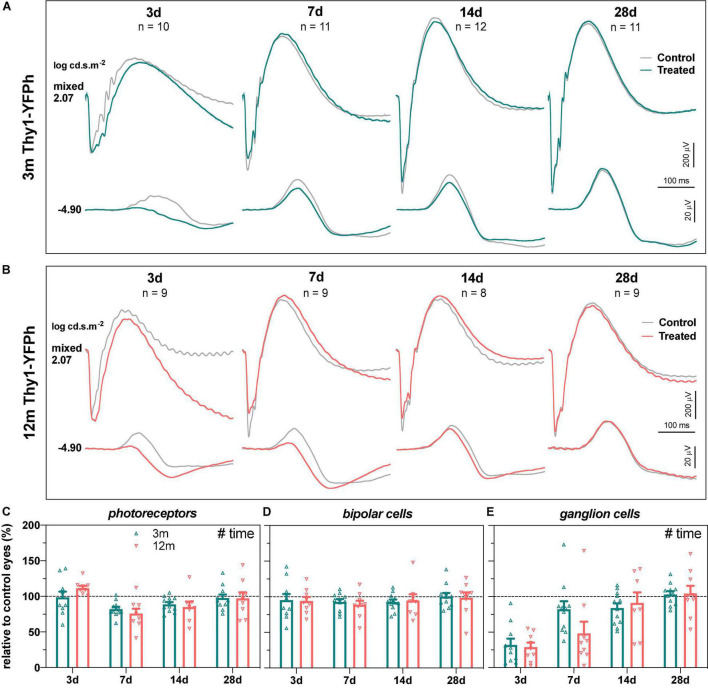
Recovery of retinal function in 3- and 12-month-old Thy1-YFPh mice. **(A)** Group averaged ERG waveforms in 3-month-old mice (teal traces, treated eyes; gray traces, control eyes) at luminous energies representing predominantly ganglion cell responses (–4.90 log cd.s.m^–2^) and outer retinal function (2.07 log cd.s.m^–2^) at 3 (*n* = 10), 7 (*n* = 11), 14 (*n* = 12), and 28 days (*n* = 11). **(B)** Group averaged ERG waveforms in 12-month-old mice (orange traces, treated eyes; gray traces, control eyes) at 3 (*n* = 9), 7 (*n* = 9), 14 (*n* = 8), and 28 days (*n* = 9). **(C–E)** ERG responses from IOP-treated eyes expressed relative to their contralateral control eyes for **(C)** photoreceptor amplitude, **(D)** bipolar cell amplitude, and **(E)** ganglion cell amplitude (pSTR) in 3-month-old (teal bars) and 12-month-old (orange bars) mice. Error bars, SEM; #, two-way ANOVA *p* < 0.05.

The functional recovery from IOP elevation in 3- and 12-month-old Thy1-YFPh mice is shown in Figure 3, for young (Figure 3A) and older eyes (Figure 3B), along with group parameters (Figures 3C–E). Consistent with C57BL/6 mice, RGC function recovered almost completely by 7 days in young Thy1-YFPh mice (Figure 3E, 82.2 ± 11.2%) but took longer (14 days) for older Thy1-YFPh mice (Figure 3E, 91.2 ± 14.7%).

**FIGURE 4:**
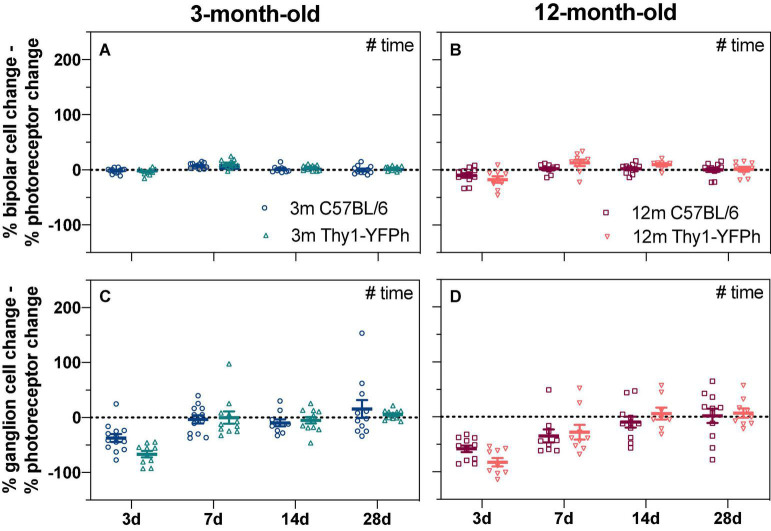
Age-matched strain comparison of bipolar cell and ganglion cell responses expressed relative to their own photoreceptor input. Responses from IOP-treated eyes were expressed relative to their contralateral control eyes (%). **(A,B)** The % photoreceptor response was subtracted from % bipolar cell response in **(A)** 3-month-old and **(B)** 12-month-old mice. This normalizes the bipolar cell response relative to the photoreceptor input. **(C,D)** The % photoreceptor response was subtracted from % ganglion cell response in **(C)** 3-month-old and **(D)** 12-month-old mice. This normalizes the ganglion cell response relative to the photoreceptor input. A resultant negative % difference indicates that ganglion cell function was more affected than expected from photoreceptor input. 3-month-old C57BL/6 mice (blue, 3 days, *n* = 13; 7 days, *n* = 12; 14 days, *n* = 10; 28 days, *n* = 11); 3-month-old Thy1-YFPh mice (teal, 3 days, *n* = 10; 7 days, *n* = 11; 14 days, *n* = 12; 28 days, *n* = 11); 12-month-old C57BL/6 mice (red, 3 days, *n* = 11; 7 days, *n* = 9; 14 days, *n* = 11; 28 days, *n* = 11); 12-month-old Thy1-YFPh mice (orange, 3 days, *n* = 9; 7 days, *n* = 9; 14 days, *n* = 8; 28 days, *n* = 9). Error bars, SEM; #, two-way ANOVA *p* < 0.05.

There were no significant strain-related differences in the way that retinal function recovered from stress, when ERG responses from IOP-treated eyes were compared to their contralateral eyes (Figure 4) or their baseline measurements (Supplementary Figure 3). This suggests that the age-related susceptibility to IOP elevation is similar in both C57BL/6 and Thy1-YFPh mice. It is worth noting that at 3 days after IOP elevation, RGC function relative to their photoreceptor input was worse in both young (Figure 4C, Thy1-YFPh -66.7 ± 5.7% vs C57BL/6 -37.3 ± 7.0%) and older (Figure 4D, Thy1-YFPh -82.5 ± 7.7% vs C57BL/6 -58.1 ± 6.1%) Thy1-YFPh mice, compared to C57BL/6 mice. Nonetheless, there was a delayed functional recovery in older mice, regardless of strains, as evident by the differences in relative pSTR responses at 7 days after IOP elevation between young and older mice.

Despite this prolonged time course for functional recovery, there were no significant changes in *in vivo* inner retinal layer thickness (measured using OCT) with time or age in either C57BL/6 or Thy1-YFPh mice (Supplementary Figure 4). Given that there were no gross structural differences, we subsequently looked for more subtle RGC structural changes may be present to mediate their functional recovery.

### Age-Related Changes in Retinal Ganglion Cells Morphology

**FIGURE 5:**
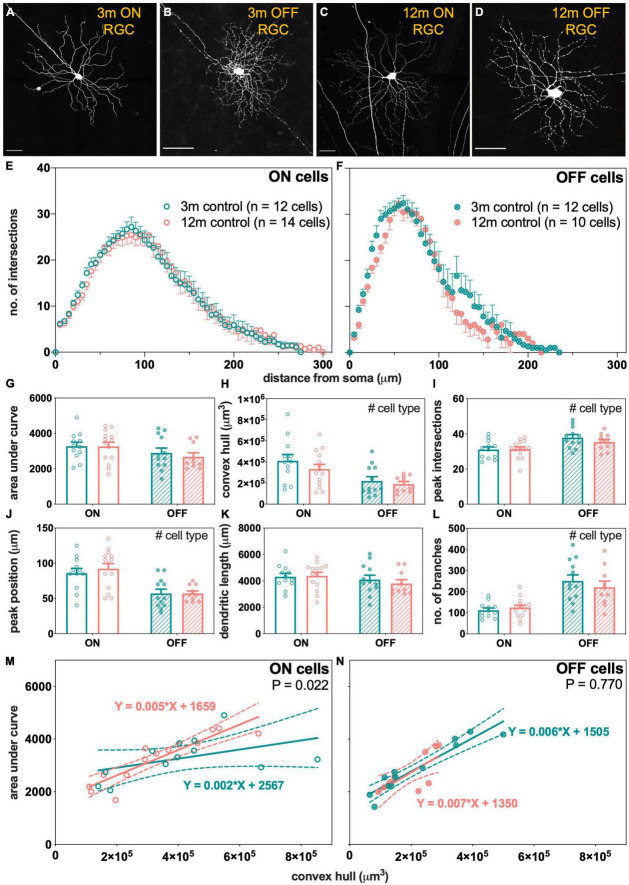
Morphological assessment of ON and OFF RGCs in 3- and 12-month-old control eyes. **(A–D)** Maximum intensity projections of confocal image z-stacks displaying dendritic arbors of representative ON and OFF RGCs in 3- and 12-month-old mice. *Scale bars*, 50 μm. **(E,F)** Sholl profiles showing group averaged dendritic complexity (no. of intersections) relative to distance from soma in **(E)** ON [3 m, teal, *n* = 12 cells (4 animals); 12 m, orange, *n* = 14 cells (3 animals)] and **(F)** OFF [3 m, teal, *n* = 12 cells (4 animals); 12 m, orange, *n* = 10 cells (4 animals)] RGCs. **(G)** Area under curve for ON (unfilled bars) and OFF (patterned bars) of 3-month-old (teal) and 12-month-old (orange) RGCs. **(H)** Convex hull volume. **(I)** Peak number of intersections. **(J)** Peak position. **(K)** Total dendritic length. **(L)** Total number of branches. **(M,N)** Dendritic complexity (area under curve) with respect to cell size (convex hull volume) in **(M)** ON and **(N)** OFF RGCs. Error bars, SEM; #, mixed effects model *p* < 0.05; solid line, linear regression; dashed line, 95% confidence interval; *p*-value, statistical significance of difference in slopes.

To understand how aging might modify IOP-induced changes to RGC morphology, normal age-related changes to RGC morphology were investigated first. RGC morphology from control eyes of 3- and 12-month-old Thy1-YFPh mice were compared. Figures 5A–D show that there were no obvious differences in the morphology of RGCs between 3- and 12-month-old ON- and OFF-RGCs. Scholl profiles show substantive overlap between ages for both ON- and OFF-RGCs (Figures 5E,F, respectively). There were no statistically significant differences between age or cell types in AUC (Figure 5G). The OFF-RGCs were smaller (Figure 5H, *p* = 0.006), had a higher peak number of intersections (Figure 5I, *p* = 0.003), had a closer peak complexity to the soma (Figure 5J, *p* < 0.001) and had more branches (Figure 5L, *p* < 0.001) compared to ON-RGCs. There were no statistically significant differences between age or cell types in total dendritic length (Figure 5K).

As RGCs were chosen at random at various retinal eccentricities and potentially contain multiple types, another way to analyze the data is to plot cell complexity [area under the Sholl curve (AUC)] against cell size (convex hull volume) for the ON- and OFF-RGCs analyzed. The slope of this function expresses how dendritic complexity changes with respect to cell size. A significant change in slope might be a more sensitive measure of changes across a heterogeneous cell group. Each data point in Figures 5M,N represents matched values of AUC and convex hull volume for each RGC analyzed. There was a statistically significant steepening of the linear relationships in 12-month-old ON cells compared to 3-month-old ON-RGCs (Figure 5M, *p* = 0.022). With increasing cell size, the dendrites of the ON-RGCs were more complex per cell volume in 12-month-old eyes compared to 3-month-old eyes. However, there was no statistically significant difference in the slopes between the two ages for OFF-RGCs (Figure 5N, *p* = 0.770).

### Change in Retinal Ganglion Cells Morphology With Intraocular Pressure Elevation in 3-Month-Old Mice

Electroretinogram findings showed that RGC function in IOP-treated eyes had recovered 7 days after injury in 3-month-old mice, but not in 12-month-old mice. In light of this, RGC morphology was compared between age groups at this recovery endpoint. Representative ON- and OFF-RGCs in control and IOP-treated eyes of 3- and 12-month-old Thy1-YFPh mice are shown in Supplementary Figure 5.

**FIGURE 6:**
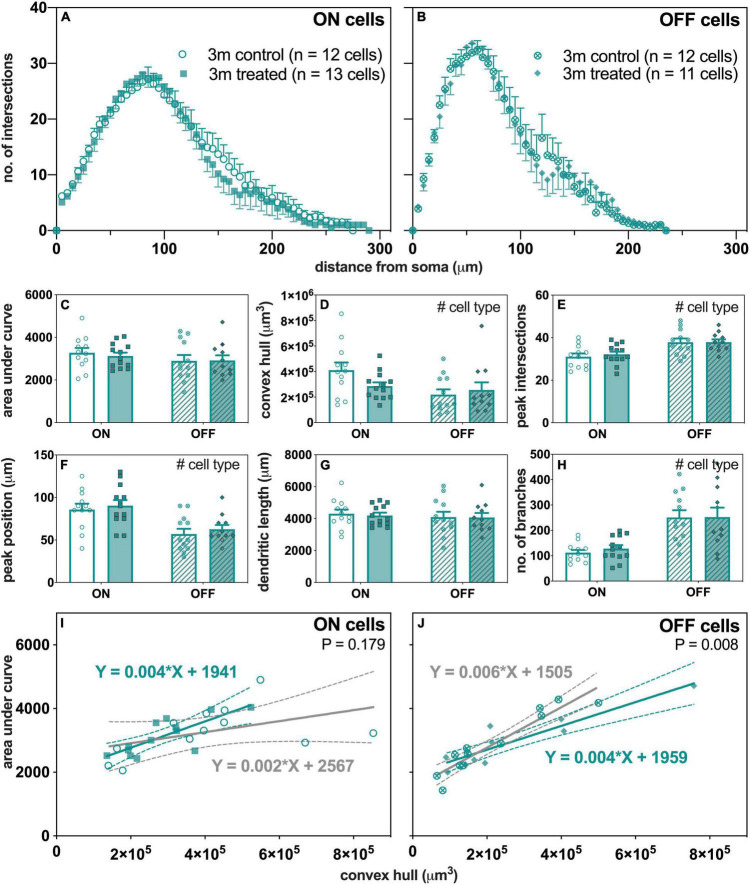
Morphological assessment of ON and OFF RGCs in 3-month-old control and IOP-treated eyes 7 days after IOP elevation. **(A,B)** Sholl profiles displaying group averaged dendritic complexity (no. of intersections) relative to distance from soma in **(A)** ON [control, unfilled circles, *n* = 12 cells (4 animals); treated, filled squares, *n* = 13 cells (4 animals) and **(B)** OFF [control, crossed circles, *n* = 12 cells (4 animals); treated, filled diamonds, *n* = 11 cells (4 animals)] RGCs. **(C)** Area under curve for control (unfilled bars) and IOP-treated (filled bars) of ON (solid bars) and OFF (patterned bars) RGCs. **(D)** Convex hull volume (# *p* = 0.021). **(E)** Peak number of intersections (# *p* < 0.001). **(F)** Peak position (# *p* < 0.001). **(G)** Total dendritic length. **(H)** Total number of branches (# *p* < 0.001). **(I,J)** Dendritic complexity (area under curve) with respect to cell size (convex hull volume) in **(I)** ON and **(J)** OFF RGCs. Error bars, SEM; #, mixed effects model *p* < 0.05; solid line, linear regression; dashed line, 95% confidence interval; *p*-value, statistical significance of difference in slopes.

Figure 6 summarizes the effect of IOP on RGC morphology in 3-month-old mice. The group averaged Sholl profiles show similar number of intersections between control and IOP-treated eyes in both ON- (Figure 6A) and OFF-RGCs (Figure 6B). There was no significant IOP effect for any of the parameters extracted from Sholl analysis, although there were the expected morphological differences between ON- and OFF-RGCs (Figures 6C–H).

When the complexity of each cell (AUC) was expressed relative to its own size (convex hull volume), there were significant linear relationships in OFF-RGCs (Figure 6J) from both control (slope 0.006, *R*^2^ = 0.89, *p* < 0.001) and IOP-treated eyes (slope 0.004, *R*^2^ = 0.84, *p* < 0.001). At 7 days after IOP injury, there was a significant flattening of the slope for OFF-RGCs (Figure 6J, *p* = 0.008), where the dendrites were less complex per cell volume. However, there was no significant difference in slopes between control and IOP-treated ON-RGCs (Figure 6I, *p* = 0.179).

### Change in Retinal Ganglion Cells Morphology With Intraocular Pressure Elevation in 12-Month-Old Mice

**FIGURE 7:**
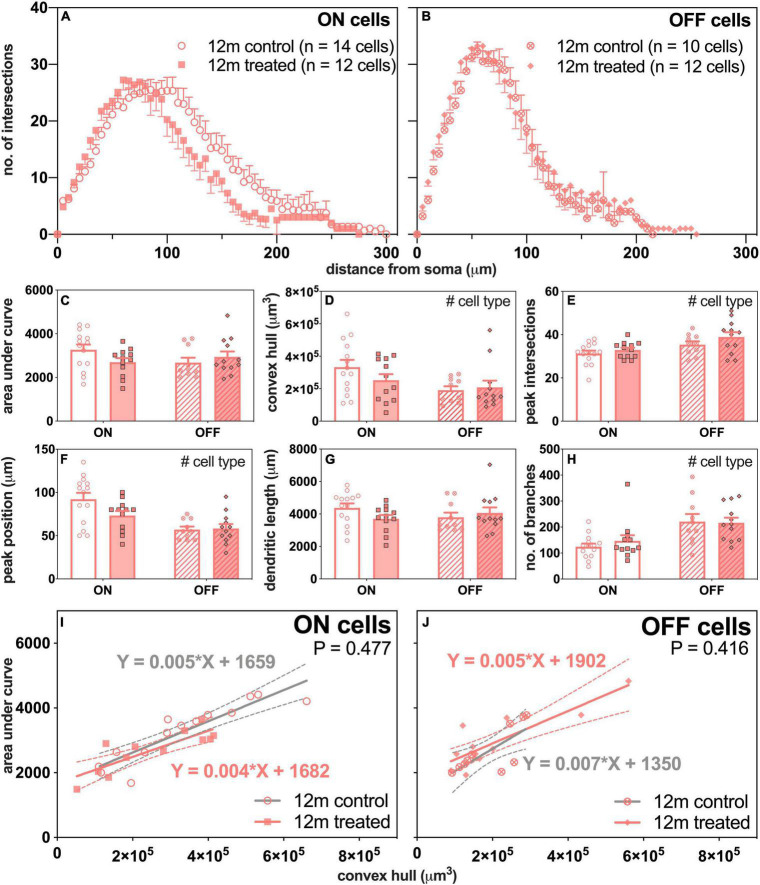
Morphological assessment of ON and OFF RGCs in 12-month-old control and IOP-treated eyes 7 days after IOP elevation. **(A,B)** Sholl profiles displaying group averaged dendritic complexity (no. of intersections) relative to distance from soma in **(A)** ON [control, unfilled circles, *n* = 14 cells (3 animals); treated, filled squares, *n* = 12 cells (4 animals)] and **(B)** OFF [control, crossed circles, *n* = 10 cells (4 animals); treated, filled diamonds, *n* = 12 cells (4 animals)] RGCs. **(C)** Area under curve for control (unfilled bars) and IOP-treated (filled bars) of ON (solid bars) and OFF (patterned bars) RGCs. **(D)** Convex hull volume (# *p* = 0.024). **(E)** Peak number of intersections (# *p* = 0.007). **(F)** Peak position (# *p* < 0.001). **(G)** Total dendritic length. **(H)** Total number of branches (# *p* < 0.001). **(I,J)** Dendritic complexity (area under curve) with respect to cell size (convex hull volume) in **(I)** ON and **(J)** OFF RGCs. Error bars, SEM; #, mixed effects model *p* < 0.05; solid line, linear regression; dashed line, 95% confidence interval; *p*-value, statistical significance of difference in slopes.

Next, we compared how ON- and OFF-RGCs responded 7 days after IOP stress in eyes of 12-month-old mice (Figure 7). There was a leftward shift in the number of intersections of the ON-RGCs in IOP-treated eyes compared to control eyes (Figure 7A). The Sholl profiles were similar in OFF-RGCs between control and IOP-treated eyes (Figure 7B).

Although there was a trend toward less complex ON-RGCs in IOP-treated compared with control eyes, overall there were no significant differences in AUC between IOP treatment or cell type (Figure 7C). Figure 7I shows significant linear relationships between AUC and convex hull volume for ON-RGCs in both control (slope 0.005, *R*^2^ = 0.81, *p* < 0.001) and IOP-treated eyes (slope 0.004, *R*^2^ = 0.70, *p* < 0.001). However, there was no significant difference in slopes between control and IOP-treated eyes (*p* = 0.477). Similarly, Figure 7J shows that for OFF-RGCs, there were significant linear relationships between AUC and convex hull volume in control (slope 0.007, *R*^2^ = 0.55, *p* = 0.014) and IOP-treated (slope 0.005, *R*^2^ = 0.73, *p* < 0.001) eyes, but again there was no significant difference between the two slopes (*p* = 0.416).

### Age-Related Changes in Retinal Ganglion Cells Morphology With Intraocular Pressure Elevation

**FIGURE 8:**
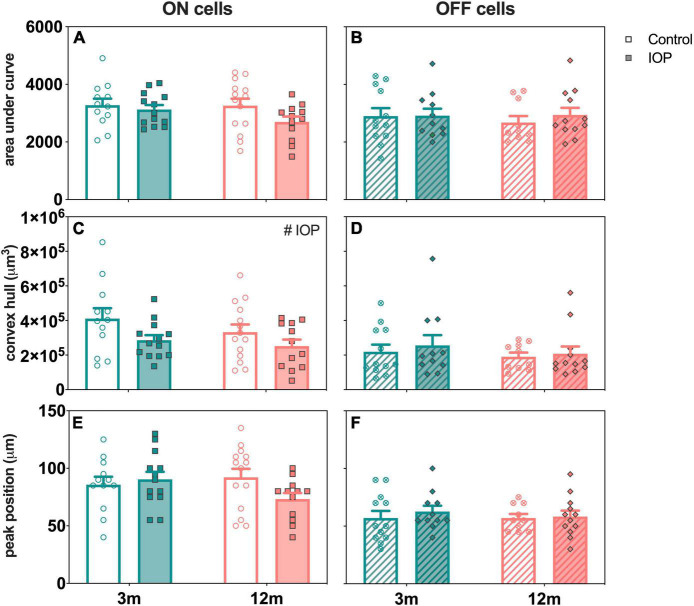
Age comparison of ON (left column) and OFF (right column) RGCs in 3- and 12-month-old eyes 7 days after IOP elevation. **(A,B)** Area under curve in 3-month-old (teal) and 12-month-old (orange) eyes for control (unfilled bars) and IOP-treated (filled bars) of **(A)** ON (solid bars) and **(B)** OFF (patterned bars) RGCs. **(C,D)** Convex hull volume. **(E,F)** Peak position. 3-month-old ON [control, *n* = 12 cells (4 animals); treated, *n* = 13 cells (4 animals)] and OFF [control, *n* = 12 cells (4 animals); treated, *n* = 11 cells (4 animals)] RGCs. 12-month-old ON [control, *n* = 14 cells (3 animals); treated, *n* = 12 cells (4 animals)] and OFF [control, *n* = 10 cells (4 animals); treated, *n* = 12 cells (4 animals)] RGCs. Error bars, SEM; #, mixed effects model *p* < 0.05.

Figure 8 directly compares the effect of age and IOP elevation on ON- (left column) and OFF-RGCs (right column). There were no statistically significant differences in AUC for both ON- and OFF-RGCs (Figures 8A,B, respectively). ON-RGCs were significantly smaller in IOP-treated compared to control eyes (Figure 8C, *p* = 0.038), however, there was no IOP effect on OFF-RGCs (Figure 8D, *p* = 0.523). Figure 8E illustrates similar Sholl profiles for ON-RGCs in 3-month-old control and IOP-treated eyes (Figure 6A), and although there was a leftward shift of Sholl profiles in 12-month-old IOP-treated eyes (Figure 7A), this was not significant (*p* = 0.084). Peak positions were similar in OFF-RGCs of both control and IOP-treated 3- and 12-month-old eyes (Figure 8F).

## Discussion

Our study showed that soon after a short period of controlled IOP elevation, RGC function showed the largest attenuation, well beyond the deficit expected due to reduced photoreceptor input (Figures 2, 4). This was most pronounced 3 days after IOP elevation with gradual recovery occurring over subsequent weeks. In particular, RGC function had fully recovered 7 days after IOP elevation in young mice and 14 days after IOP elevation in older mice. Given the age-related difference in recovery at the 7-day endpoint, RGC morphology was examined. Both young and older ON-RGCs were smaller in IOP-treated compared to contralateral control eyes (Figure 8C). In 3-month-old mice, OFF-RGC dendrites in IOP-treated eyes became less complex relative to their cell size compared to control eyes (Figure 6J). Changes in RGC dendritic complexity relative to cell size was not observed in 12-month-old mice (Figures 7I,J).

### Recovery of Retinal Function After Intraocular Pressure Elevation

Consistent with previous rodent studies, we find that older eyes showed greater functional deficits than younger eyes in response to acute IOP elevation (Kong et al., 2012; Charng et al., 2013; Lim et al., 2014; Zhao et al., 2018). Seven days after IOP elevation, although RGC function had largely recovered in 3-month-old mice (82 ± 11%), there was still dysfunction in 12-month-old mice (64 ± 8%; Kong et al., 2009, 2012). RGC function in 12-month-old mice did not return to normal levels until 28 days post IOP injury (Crowston et al., 2017; Fry et al., 2018). Our findings at the 7-day recovery endpoint are consistent with the literature for both young and older mice. We extend previous studies to show that between 7 and 14 days, younger eyes had recovered from 50 mmHg of IOP elevation, whereas older eyes were still undergoing recovery.

Interestingly, at the 3-day recovery endpoint in both age groups, RGC responses (relative to their photoreceptor input) in Thy1-YFPh mice were poorer compared to those in age-matched C57BL/6 mice (Figure 4C, 3 m; Figure 4D, 12 m). However, when RGC responses were expressed relative to their contralateral control eyes, recovery at 3 days after IOP elevation was similar in both age groups in both strains [3 m, C57BL/6 mice (Figure 1E) and Thy1-YFPh mice (Figure 3E); 12 m, C57BL/6 mice (Figure 1E) and Thy1-YFPh mice (Figure 3E)]. A comparison of outer retinal ERG responses in control eyes at endpoints expressed relative to their own baselines (untreated eyes) showed significantly smaller responses in young Thy1-YFPh mice 3 days after cannulation (Supplementary Figure 6). These findings suggest that Thy1-YFPh mice may be more sensitive to repeated anesthesia than C57BL/6 mice, such that when anesthesia was re-administered 3 days after cannulation, ERG responses were attenuated in both control and cannulated eyes. Nonetheless, this effect appeared to have recovered by 7 days after the previous anesthesia, as RGC functional recovery was similar between C57BL/6 and Thy1-YFPh mice.

### Age-Related Changes to Retinal Ganglion Cells Morphology

Morphological assessment showed that older ON-RGCs were relatively more complex as a function of increasing cell size compared to younger ON-RGCs (Figure 5M). However, it is worth noting that overall OFF-RGCs were significantly smaller than ON-RGCs in both age groups (Figure 5H) and the range of OFF-RGCs sampled in older eyes was narrower (0.93 × 10^5^–2.90 × 10^5^ μm^3^) than younger OFF-RGCs (0.65 × 10^5^–5.00 × 10^5^ μm^3^; Figure 5N). It is less likely that the narrower range of OFF-RGCs was due to cell loss during normal aging as there was no significant cell nuclei reduction in the ganglion cell layer between 2 to 12 months of age (Ferdous et al., 2021). Nonetheless, this narrower range of OFF-RGCs may have limited our capacity to find differences in OFF-RGCs with age.

Data in humans and animal models suggest that aging results in smaller RGCs. RGCs showed reductions in dendritic areas, dendritic field coverage, branch points, terminal neurite tips and Sholl area in melanopsin-expressing RGCs in healthy donor retina of those aged 50 years and above compared to those under 30 years of age (Esquiva et al., 2017). Samuel et al. (2011) reported significant shrinkage of dendritic field areas, less retinal coverage, and reduction in inner plexiform layer synaptic density for ON- and OFF-RGCs in 24- to 28-month-old mice compared to 3- to 5-month-old mice. Despite these structural differences, the authors showed that single cell RGC responses to light were preserved in older mice (Samuel et al., 2011). That RGC function remained normal despite these morphological changes could reflect the possibility that (1) these morphological changes have not exceeded functional redundancy or (2) more subtle compensatory adaptations, such as RGC dendritic remodeling or synaptic rearrangement, may have occurred.

We found some evidence of subtle adaptation as evident by a steepening of the slope describing the relationship between dendritic complexity of ON RGCs against cell size in 12-month-old compared with 3-month-old RGCs (Figure 5M), which may reflect RGC adaptations to normal aging. Why ON-RGCs might show more prominent age-related changes may lie in their higher energy demand compared with OFF-RGCs (Williams et al., 2010, 2012). There were selective reductions in postsynaptic density counts in sublamina-a (synapses with ON-RGCs), dendritic fields and total dendritic lengths in older *Opa1*^±^ mice (mitochondrial dysfunction; Williams et al., 2010, 2012). In this regard, studies of age-related changes in excitatory and inhibitory synaptic relationships might provide insights into age-related RGC functional adaptations.

### Intraocular Pressure Effects on ON- and OFF-Retinal Ganglion Cells Morphology

We found that ON-RGCs showed reduced cell size with IOP elevation in both young and older mice (Figure 8C). Some studies reported reduced dendritic field areas (Feng et al., 2013, 2016; Risner et al., 2018) while others found no significant changes (Della Santina et al., 2013; El-Danaf and Huberman, 2015; Ou et al., 2016). Along the same lines, some studies showed reduced ON-RGC dendritic complexity (Ou et al., 2016; Risner et al., 2018; Bhandari et al., 2019), but others reported no significant changes (Della Santina et al., 2013; El-Danaf and Huberman, 2015). Besides that, Risner et al. (2018) showed reduced dendritic complexity, dendritic field area and dendritic length in ON-, OFF-, and ON-OFF RGCs in DBA/2J mice. We did not find a change in ON-RGC complexity, which may be related to our shorter IOP stressor compared to chronic IOP elevation in other studies.

Of interest, better functional recovery in young mice was associated with IOP-induced changes in OFF-RGC dendrites (Figure 6J). However, there was no significant change in OFF-RGC dendritic complexity in older eyes (Figure 7J). This argues that morphological dendritic remodeling is a beneficial adaptive response, perhaps to facilitate more rapid functional recovery from an IOP stress. Data from Zhao et al. (2019) showed that there was very little additional loss of RGCs between 8 and 12 weeks of chronic IOP elevation using the circumlimbal suture model, although functional recovery was possible if IOP was normalized after 8 weeks, but not after 12 weeks of IOP elevation (Zhao et al., 2019). This observation would suggest that the loss of functional recovery capacity after 12 weeks of IOP elevation was not because more RGCs were lost, but more likely due to a decline in the capacity of the remaining RGCs to recover. Whether this failure to recover is related to a loss of RGCs intrinsic capacity for morphological remodeling would be worthy of investigation.

Our finding that a short period of IOP stress changed dendritic morphology in OFF-RGCs (less complex relative to cell size) could be interpreted to agree with the greater sensitivity of OFF-RGCs observed in other studies (Della Santina et al., 2013; El-Danaf and Huberman, 2015; Ou et al., 2016), even when axon degeneration was delayed *via* protection from the slow *Wallerian degeneration* (*Wld^S^*) allele (Risner et al., 2021). Comparison across age groups would suggest that OFF-RGC adaptations may aid functional recovery, as there was no OFF-RGC remodeling in older eyes. Thus, these morphological changes could be seen as an adaptive and beneficial response to stress. A corollary of this is that it appears that older RGCs have less capacity to remodel their dendrites compared to younger cells when exposed to the same IOP stressor. Why this capacity for remodeling in OFF-RGCs is lost in older eyes is unclear. Further investigations to study whether dendritic remodeling is delayed and would be evident at a later time following RGC functional recovery from IOP elevation (i.e., after 14 or 28 days) in older mice would be useful in understanding how aging changes the adaptive mechanism.

In contrast to the age-related difference in OFF-RGC morphological response to IOP elevation, ON-RGCs in both young and older eyes showed a reduction in cell size. As ON-RGCs are known to be more metabolically demanding (Williams et al., 2010, 2012), one might speculate that a reduction in cell size after IOP stress could aid recovery by reducing energy demand. There may also be adaptations at the level of excitatory and inhibitory inputs. Some studies have shown reduced excitatory postsynaptic densities and reduced spontaneous and light-evoked firing rates of RGCs in response to IOP elevation in young mice (Della Santina et al., 2013; Ou et al., 2016). Ou et al. (2016) also reported loss of presynaptic ribbon density in the OFF-sublamina after transient IOP elevation in young mice. This may be related to our finding that OFF-RGC dendrites became significantly less complex as a function of increasing size in young mice. Whether changes in synapses and firing rates are differentially altered after IOP elevation in older mice has yet to be investigated.

Overall, functional recovery from IOP elevation in Thy1-YFPh mice from IOP stress was largely similar to the widely used C57BL/6 mice. This study has a number of limitations. Firstly, previous studies have shown that IOP elevation may lower Thy1 expression in RGCs (Huang et al., 2006; Williams et al., 2013; Crowston et al., 2015), thus the changes in RGC morphology reported here need to be interpreted with caution. Our finding of greater OFF-RGC sensitivity to IOP elevation is consistent with other studies using alternate approaches for morphological analyses (El-Danaf and Huberman, 2015; Ou et al., 2016). These findings suggest that the YFP expression was a robust marker for assessing RGC morphology, otherwise one would have expected a more uniform downregulation of Thy1 in both ON- and OFF-RGCs following IOP elevation. Nevertheless, other approaches would be useful to confirm this, including intracellular dye filling, which if used on the fluorescent RGCs in Thy1-YFPh mice would differentiate true changes in morphology from Thy1 downregulation. Besides that, although we did not measure IOP at each endpoint, our previous experience showed that there were no significant age-related differences in baseline IOP between 3 and 12 months (Supplementary Figure 7). As our acute IOP elevation model increases IOP for a duration of 30 min after which IOP returns to baseline levels, we did not expect significant age-related IOP differences at each endpoint. Furthermore, we acknowledge that our analysis of the different reactions of RGC types on IOP-related stress is limited to two broad categories based on their dendritic stratification in the inner plexiform layer. Additional sub-classification including functional characterization of each cell type would provide significant insight toward understanding age-related RGC type-specific vulnerability to IOP elevation.

## Conclusion

Following an acute reversible IOP elevation to 50 mmHg for 30 min, RGC function fully recovered by 7 days in young mice and 14 days in older mice. The 7-day recovery endpoint provided a useful window to examine age-related changes to RGC morphology. We showed that in healthy aging, the ON-RGC dendrites became more complex per cell volume compared to their younger counterparts. The ON-RGCs became significantly smaller in cell volume in both age groups 7 days after IOP elevation. Better functional recovery was associated with the capacity for morphological change in OFF-RGCs in young mice, but not older mice. These findings show that there is an age-related RGC type-specific susceptibility in response to an IOP stress, providing insights into how aging affects their functional recovery.

## Data Availability Statement

The raw data supporting the conclusions of this article will be made available by the authors, without undue reservation.

## Ethics Statement

The animal study was reviewed and approved by The Florey Institute of Neuroscience and Mental Health Animal Ethics Committee.

## Author Contributions

PL and BB: study design, data collection, data analysis, and manuscript preparation. DZ and VW: data collection and manuscript preparation. VC and JC: study design and manuscript preparation. All authors contributed to the article and approved the submitted version.

## Conflict of Interest

The authors declare that the research was conducted in the absence of any commercial or financial relationships that could be construed as a potential conflict of interest.

## Publisher’s Note

All claims expressed in this article are solely those of the authors and do not necessarily represent those of their affiliated organizations, or those of the publisher, the editors and the reviewers. Any product that may be evaluated in this article, or claim that may be made by its manufacturer, is not guaranteed or endorsed by the publisher.
