# Supplementary material for: The Effect of Aging on Retinal Function and Retinal Ganglion Cell Morphology Following Intraocular Pressure Elevation
Source: Front Aging Neurosci. 2022 May 12;14:859265. doi: 10.3389/fnagi.2022.859265 (PMC9133539; doi:10.3389/fnagi.2022.859265)
Supplement: Supplementary file 1 [file Data_Sheet_1.pdf]

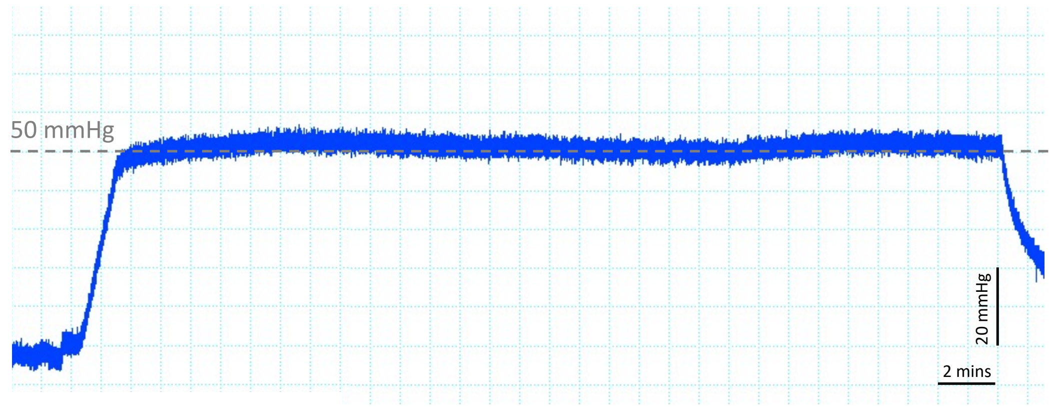

Supplementary Figure 1 A representative example of real time IOP tracking on the LabChart software.

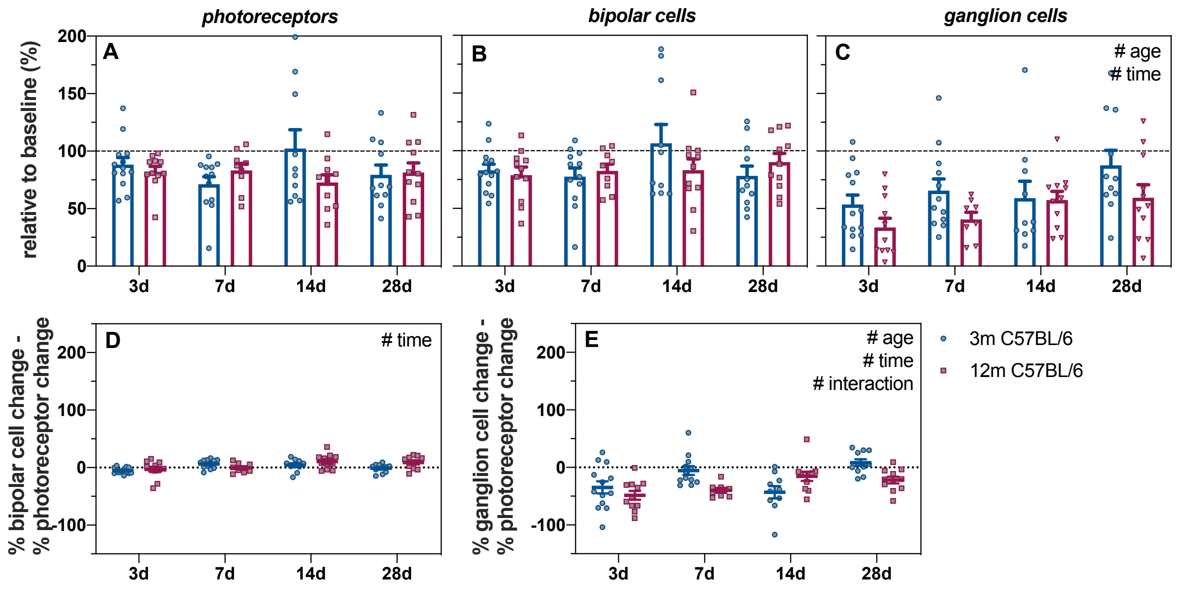

Supplementary Figure 2 Recovery of retinal function in 3- and 12-month-old C57BL/6 mice, expressed relative to baseline measurements. (A – C) ERG responses from IOP-treated eyes expressed relative to their baseline measurements for (A) photoreceptor amplitude, (B) bipolar cell amplitude and (C) ganglion cell amplitude in 3-month-old (blue bars) and 12-month-old (red bars) mice. **(D – E)** Age comparison of (D) bipolar cell and (E) ganglion cell responses expressed relative to their own photoreceptor input. *Error bars, SEM; #, two-way ANOVA $p < 0.05$.*

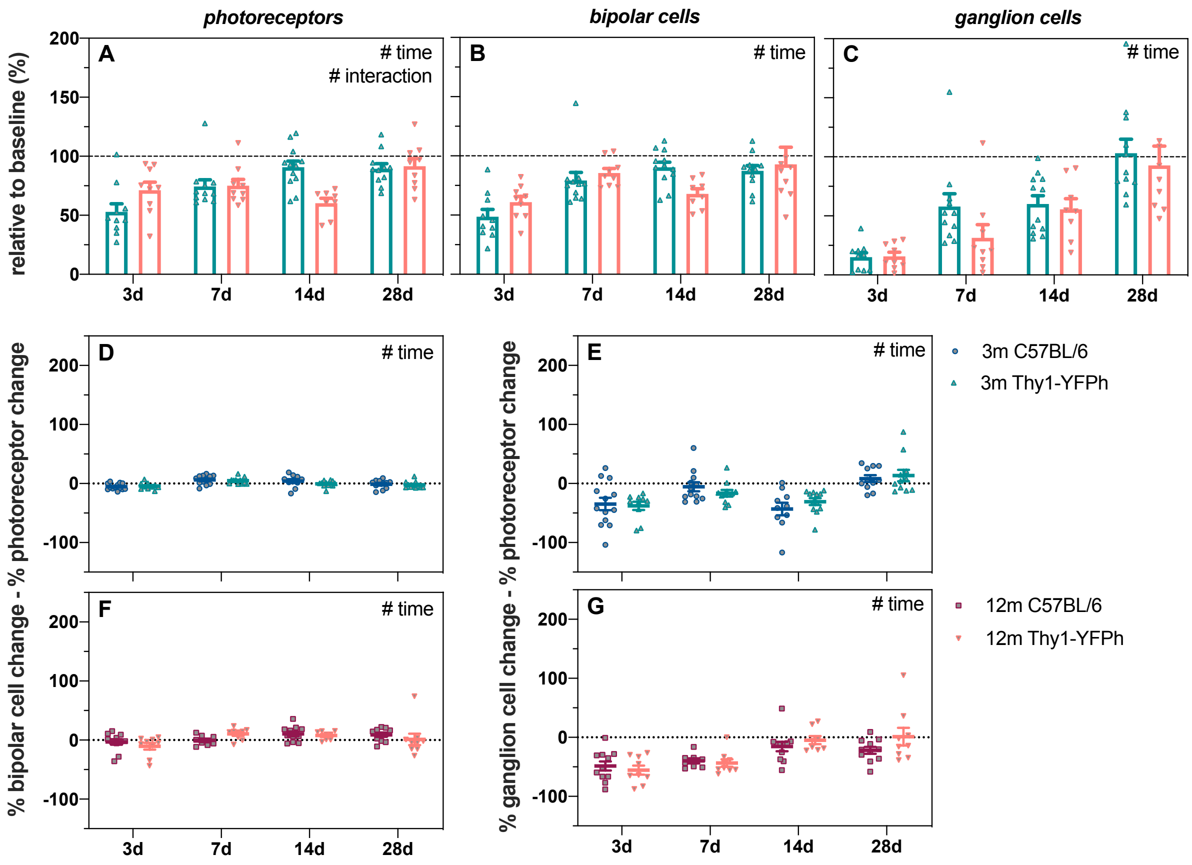

Supplementary Figure 3 Recovery of retinal function in 3- and 12-month-old Thy1-YFPh mice, expressed relative to baseline measurements. (A – C) ERG responses from IOP-treated eyes expressed relative to their baseline measurements for (A) photoreceptor amplitude, (B) bipolar cell amplitude and (C) ganglion cell amplitude in 3-month-old (blue bars) and 12-month-old (red bars) mice. **(D – E)** Strain comparison of bipolar cell and ganglion cell responses expressed relative to their own photoreceptor input in 3-month-old (D and E, respectively) and 12-month-old (F and G, respectively) mice. *Error bars, SEM; #, two-way ANOVA p < 0.05.*

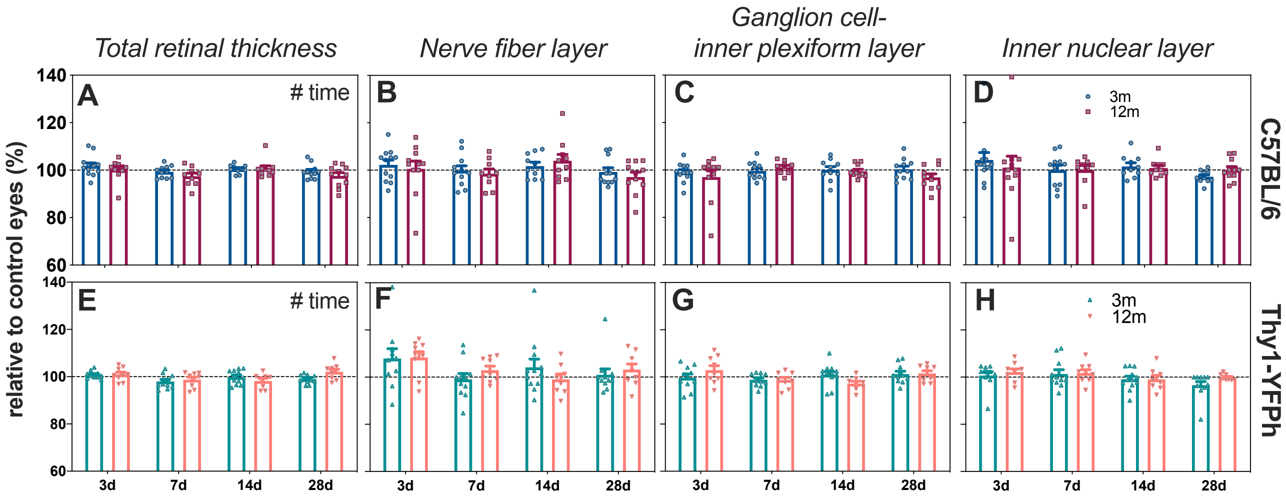

Supplementary Figure 4 Age comparison of relative inner retinal layer thicknesses measured using optical coherence tomography (OCT) in 3- and 12-month-old (A – D) C57BL/6 and (E – H) Thy1-YFPh. (A & E) Total retinal thickness. **(B & F)** Nerve fiber layer. **(C & G)** Ganglion cell – inner plexiform layers. **(D & H)** Inner nuclear layer. 3-month-old C57BL/6 mice (blue, 3 days, n = 12; 7 days, n = 11; 14 days, n = 10; 28 days, n = 11); 3-month-old Thy1-YFPh (teal, 3 days, n = 10; 7 days, n = 11; 14 days, n = 12; 28 days, n = 11); 12-month-old C57BL/6 (red, 3 days, n = 11; 7 days, n = 9; 14 days, n = 10; 28 days, n = 11); 12-month-old Thy1-YFPh (orange, 3 days, n = 9; 7 days, n = 9; 14 days, n = 8; 28 days, n = 9). *Error bars, SEM; #, two-way ANOVA $p < 0.05$; black dashed line, 100% indicating no difference between treated and control eyes.*

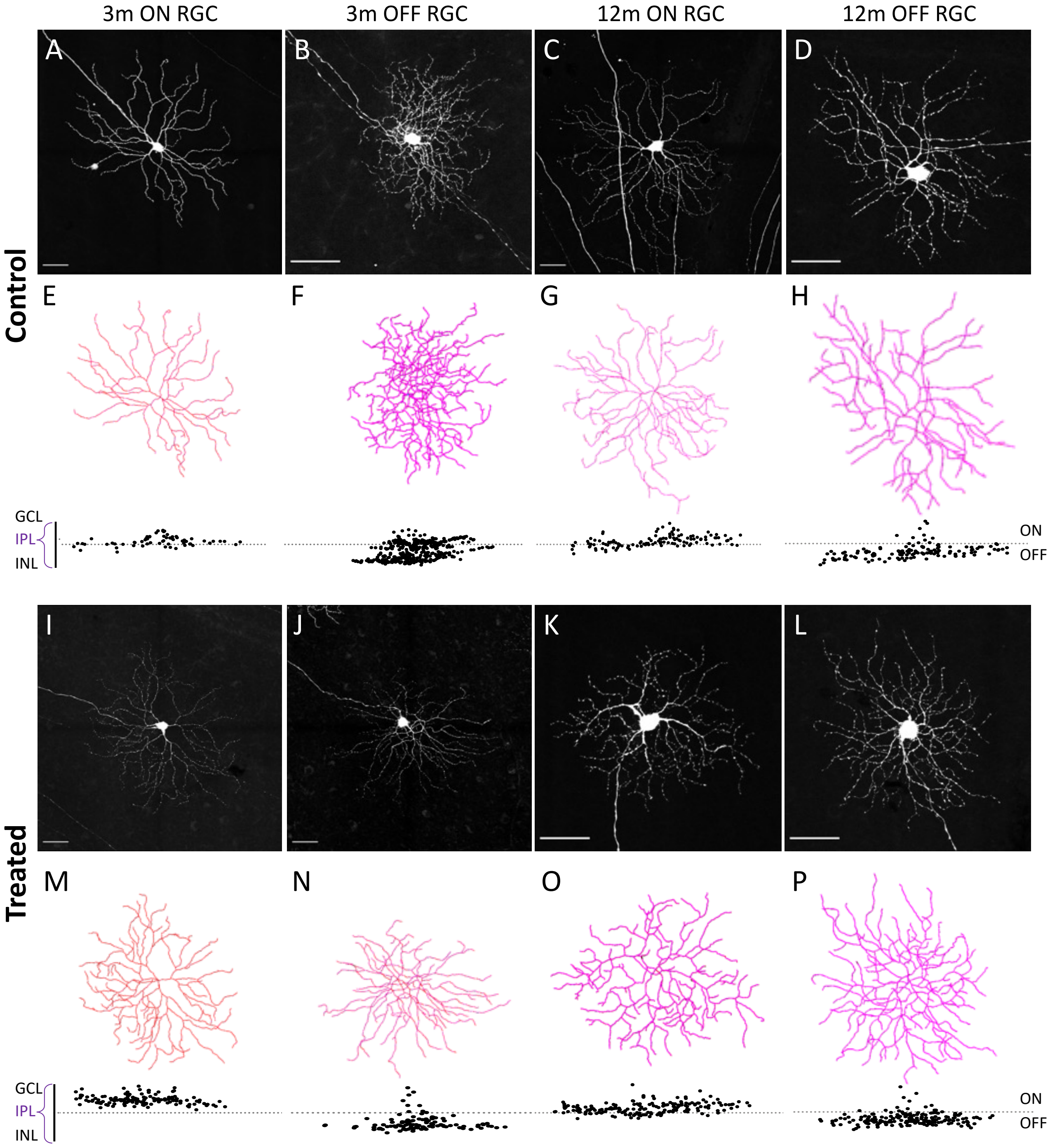

Supplementary Figure 5 Dendritic arbors of ON and OFF RGCs in 3- and 12-month-old (A – D) control and (I – L) IOP-treated eyes. (E – H) The corresponding skeletonized images of Panels A – D, and the position of the dendrites in the inner plexiform layer. **(M – P)** The corresponding skeletonized images of Panels I – L, and the position of the dendrites in the inner plexiform layer. *Scale bars, 50 μm; GCL, ganglion cell layer; IPL, inner plexiform layer; INL, inner nuclear layer; grey dotted line, 50% mark of the IPL.*

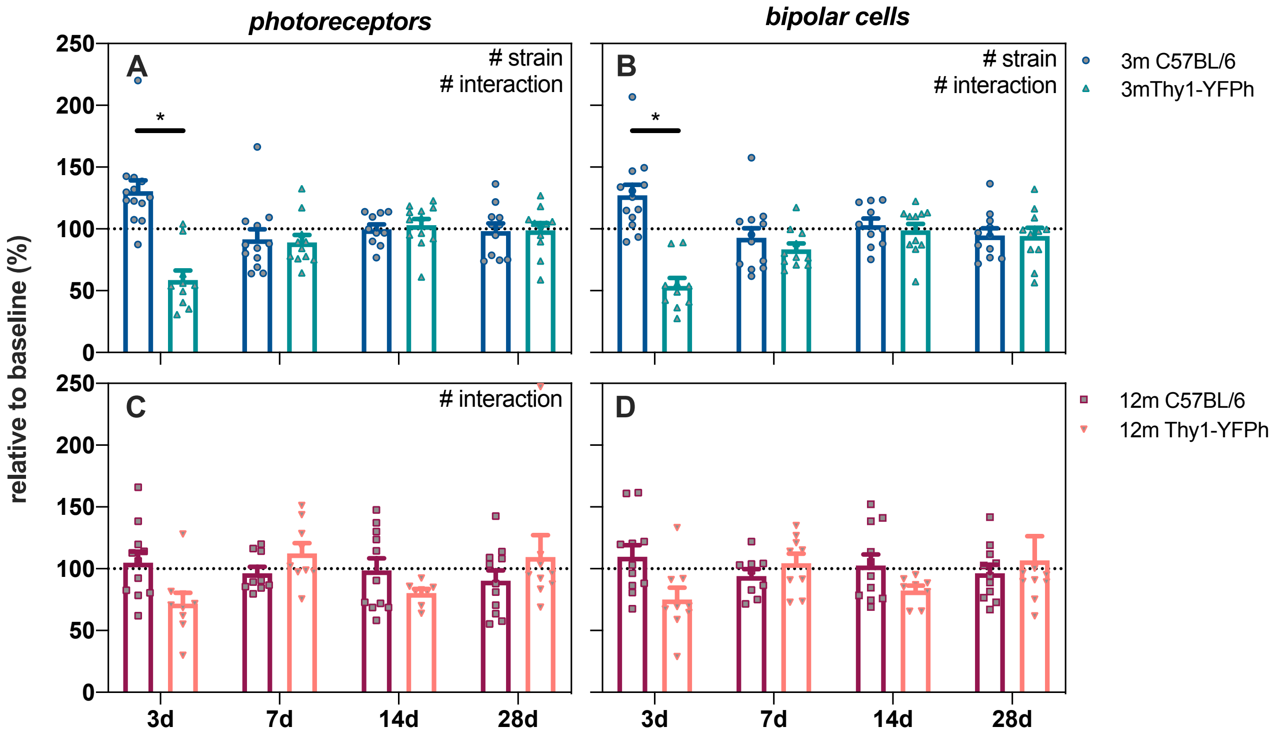

Supplementary Figure 6 Age-matched strain comparison of photoreceptor and bipolar cell responses in contralateral control eyes relative to their own baseline measurements. (A & B) Relative (A) photoreceptor amplitude and (B) bipolar cell amplitude in 3-month-old C57BL/6 (blue) and Thy1-YFPh (teal) mice. **(C & D)** Relative (C) photoreceptor amplitude and (D) bipolar cell amplitude in 12-month-old C57BL/6 (red) and Thy1-YFPh (orange) mice. *Error bars, SEM; #, two-way ANOVA $p < 0.05$; *, Sidak's multiple comparison test $p < 0.05$.*

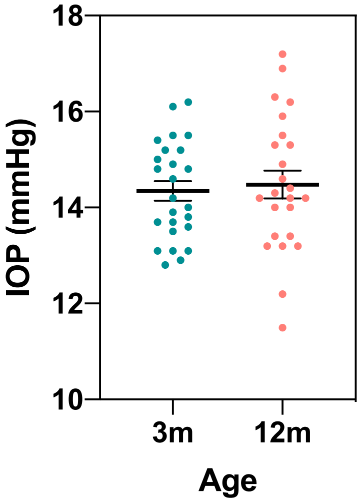

Supplementary Figure 7 Age comparison of baseline IOP measurements of 3-month-old (teal, n = 25, 14.3 ± 0.2 mmHg) and 12-month-old (orange, n = 24, 14.5 ± 0.3 mmHg) Thy1-YFPh mice. There was no statistically significant difference between age (two-tailed t-test, p-value 0.70). *Error bars, SEM.*
